# Venus Kinase Receptors: Prospects in Signaling and Biological Functions of These Invertebrate Kinases

**DOI:** 10.3389/fendo.2014.00072

**Published:** 2014-05-13

**Authors:** Colette Dissous, Marion Morel, Mathieu Vanderstraete

**Affiliations:** ^1^INSERM U1019, CNRS-UMR 8204, Center for Infection and Immunity of Lille, Institut Pasteur de Lille, Université Lille Nord de France, Lille, France

**Keywords:** venus kinase receptor, structure, phylogeny, kinase signaling, function, reproduction

## Abstract

Venus kinase receptors (VKRs) form a family of invertebrate receptor tyrosine kinases (RTKs) initially discovered in the parasitic platyhelminth *Schistosoma mansoni*. VKRs are single transmembrane receptors that contain an extracellular venus fly trap structure similar to the ligand-binding domain of G protein-coupled receptors of class C, and an intracellular tyrosine kinase domain close to that of insulin receptors. VKRs are found in a large variety of invertebrates from cnidarians to echinoderms and are highly expressed in larval stages and in gonads, suggesting a role of these proteins in embryonic and larval development as well as in reproduction. *VKR* gene silencing could demonstrate the function of these receptors in oogenesis as well as in spermatogenesis in *S. mansoni*. VKRs are activated by amino acids and are highly responsive to arginine. As many other RTKs, they form dimers when activated by ligands and induce intracellular pathways involved in protein synthesis and cellular growth, such as MAPK and PI3K/Akt/S6K pathways. VKRs are not present in vertebrates or in some invertebrate species. Questions remain open about the origin of this little-known RTK family in evolution and its role in emergence and specialization of Metazoa. What is the meaning of maintenance or loss of VKR in some phyla or species in terms of development and physiological functions? The presence of VKRs in invertebrates of economical and medical importance, such as pests, vectors of pathogens, and platyhelminth parasites, and the implication of these RTKs in gametogenesis and reproduction processes are valuable reasons to consider VKRs as interesting targets in new programs for eradication/control of pests and infectious diseases, with the main advantage in the case of parasite targeting that VKR counterparts are absent from the vertebrate host kinase panel.

## Introduction

Tyrosine kinases (TKs) belong to the eukaryotic protein kinase (ePK) superfamily. By catalyzing the transfer of phosphate groups on tyrosine residues, these enzymes induce changes in the conformation and/or the activity of their protein substrates. TKs play essential functions in fundamental cellular processes such as proliferation, differentiation, or migration (1) and are divided into two major groups: cytoplasmic TKs (CTKs) and membrane receptor tyrosine kinases (RTKs). RTKs transduce specific extracellular signals through the cell membrane and initiate the intracellular phosphotyrosine signal, which is relayed by various proteins including CTKs (2, 3).

Tyrosine kinases constitute an ancient protein family found in unicellular organisms such as Choanoflagellates, Ichthyosporeans, and Corallochytreans (4, 5). Interestingly, although CTK repertoires are well conserved from unicellular to metazoan species, RTK sets have evolved differently (5). In humans, 90 genes encode TK proteins. They represent 15% of the protein kinome and among these TKs, 58 are RTKs which have been grouped into 20 classes, based on the sequences of their kinase domains (6, 7). Such a clustering closely parallels their overall structure and the specificity of their extracellular-binding domains. Some of the RTK classes (particularly the class I with epidermal growth factor receptor, EGFR, and the class II with insulin receptors, IR/IGFR) were found across all metazoan lineages, including Radiata (sponges and jellyfishes) and receptor sequences were relatively well conserved (8). However, global RTK class distribution is highly variable throughout evolution and RTK profiles are different in the various animal phyla (9). Even more, RTKs can exist, which are specific for one species, such as the sweet tooth receptor of *Hydra vulgaris* (10) or kin15/kin16 proteins of *Caenorhabditis elegans* (11).

Venus kinase receptors (VKRs) constitute a unique family of RTKs, exclusively present in invertebrate species. VKRs have a typical RTK structure, composed of an intracellular TK domain linked to an extracellular moiety by a unique transmembrane domain and like many RTKs they are active as dimers formed at the cell membrane (2). From the homology of their TK domains with those of insulin receptors, VKRs would have been *a priori* sorted with class II RTKs, but the finding of their special extracellular domain led us to attribute to them a novel identity with the name of “VKR” (12). Indeed, the VKR receptor uses a venus fly trap (VFT) structure as ligand-binding domain which is required for kinase activation of the receptor. VFT modules are large domains composed of two lobes separated by a highly flexible cleft, allowing the binding of a small molecule. They are found in various membrane proteins from bacteria (such as periplasmic binding proteins) to higher metazoans where they constitute the ligand-binding domains of many receptors like the class C G protein-coupled receptors (GPCRs), sweet taste receptors and atrial natriuretic factor receptors (ANFRs) (13, 14). For this reason, VFT receptors are the targets of many psychotropic drugs in human medicine.

**Figure 1:**
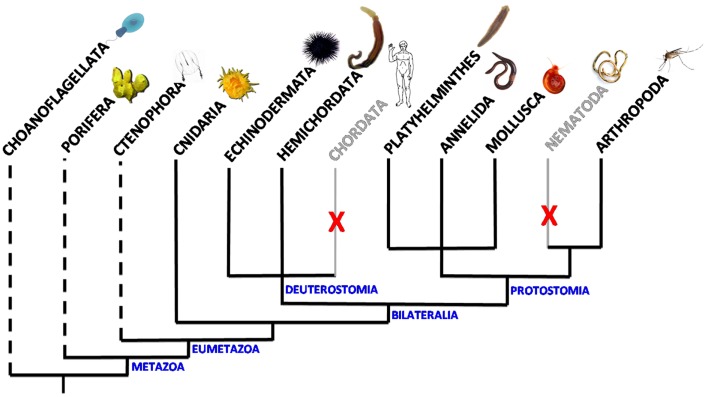
**Scheme of the distribution of VKR in animal phyla**. *VKR* genes are present and expressed in Cnidaria, Arthropoda, Platyhelminths, Annelida, Mollusca, Hemichordata, and Echinodermata phyla. They were not found in the genomes of chordates and nematodes. Dashed lines indicate that the presence of VKR is speculative in these phyla due to the lack of genomic data. However, putative RTKs similar to VKR have been already detected in the choanoflagellates *Monosiga brevicollis* (18) and *Salpingoeca rosetta* (19).

Venus kinase receptors were discovered for the first time in the platyhelminth *Schistosoma mansoni* (15). Searches in available genomic databases further indicated that *VKR* genes were part of a large variety of genomes, all of invertebrates, and their expression was confirmed in seven distinct phyla: Cnidaria, Arthropoda, Platyhelminthes, Annelida, Mollusca, Hemichordata, and Echinodermata. The presence of VKR in the cnidarian *Nematostella vectensis* suggests that the family appeared with the emergence of eumetazoan species (12, 16) (Figure 1). Putative RTKs with an architecture close to that of VKR proteins have been found in choanoflagellates. Accordingly, the existence of VKR in free-living unicellular and colonial flagellates lets suppose that it might have contributed to the establishment of multicellularity (16). Among the 55 VKRs discovered to date, most of them (31 sequences) were found in arthropods, all belonging to the Hexapoda subphylum and to the class of insects. Complete VKR sequences could not still be detected in the other subphyla of Arthropoda (Chelicerata, Myriapoda, and Crustacea), even if a truncated sequence that might correspond to VKR has been noted in the genome of the water flea *Daphnia pulex*. Insect *VKR* genes were identified in Diptera (*Drosophila* flies and mosquitoes) as well as in Coleoptera, Hymenoptera and *Phthiraptera* orders. Interestingly, in addition to the finding that *VKR* genes were absent from several major arthropod subphyla, it was observed that inside of the *Drosophila* genus several species, belonging particularly to the *melanogaster* group, are also missing *VKR* genes. Similarly, in helminths, *VKR* genes were found only in species belonging to the platyhelminth phylum, i.e., in trematodes and cestodes, but they were not identified in nematodes (16). Therefore, *VKR* genes are unfortunately absent from the two invertebrate models *C. elegans* and *Drosophila melanogaster* which offer highly efficient tools for genetics*. VKR* genes are found as single copies in most species, except in some insects (lepidopterans) and in platyhelminths in which two different *VKR* copies are present. Copies were found either in tandem on the same scaffold (lepidopterans) or on distinct chromosomes (trematodes). In *S. mansoni*, the two gene copies have a quite identical exon–intron organization, and this argues for a duplication event in this trematode parasite (17).

Studies of exon–intron composition have shown a wide heterogeneity of *VKR* genes across the diverse phyla. *VKR* genes found in lophotrochozoan organisms (annelids, mollusks, platyhelminths) are overall more complex (15–18 exons) than the insect ones (5 exons in flies and mosquitoes), and more similar to that detected in the basal cnidarian *N. vectensis* (15 exons) or to *Spvkr* found in the echinoderm *Strongylocentrotus purpuratus* (sea urchin). However, despite their important heterogeneity in size and complexity, *VKR* genes possess common features in the genomic regions encoding the functional protein domains that are conserved from Cnidaria to all other phyla. This shows that *VKR* genes definitely encode a novel RTK family, widespread in the bilaterian branch of *Eumetazoa*, with possibly an origin close in time to that of the setting-up of animal multicellularity (16). This little-known RTK family deserved to be further explored in order to determine more precisely its evolutionary origin, its possible importance for the emergence and specialization of Metazoa, and to understand how its maintenance or its loss in various phyla or in some species could be in relation with development and physiological functions.

## Structure and Activation of VKR

Venus kinase receptors are true RTKs with a conserved TK catalytic domain. Phylogenetic relationships between the TK domains from VKRs and from various RTKs (IR, EGFR, and ROS) showed that all VKR TK domains formed a monophyletic group close to TK domains of IRs (12) and further results obtained from the alignment of the full-length receptor sequences confirmed the proximity between VKRs and IRs (16, 17). As it can be expected for catalytic structures, TK domains are well conserved across all species, with the best scores of identity observed between the species belonging to a same order. In most of the TK domains from VKRs, the motifs crucial for TK activity are found, such as the ATP binding site (GXGXXG), the sequence required for ATP stabilization (VAVKX_16_E), the catalytic loop implicated in phosphotransfer (HRDXAXRN), the Mg^2+^ binding site (DFG), the consensus PVRWMXPE sequence considered as a strong indicator of tyrosine substrate specificity, and the two putative autophosphorylation sites (YY) allowing an open access to ATP and substrates in many activated RTKs, including IRs (20, 21). Furthermore, it was demonstrated that several recombinant VKRs of insects and trematodes are able to autophosphorylate and to induce signaling when expressed in heterologous cells, indicating that VKRs were active kinases and functional RTKs (17, 22).

Venus fly trap modules of VKR proteins were compared with those of known VFT-containing receptors including class C GPCRs [mGluR, GABABR1/2, and CaS (Calcium-sensing) receptors], receptors with guanylate cyclase activity (ANFR) and NMDA ionotropic glutamate receptor (iGluR), all these receptors being present in invertebrates (from cnidaria to insects) and in vertebrates. Phylogenetic data obtained from this alignment showed that all VKR proteins formed a single group closely related to class C GPCRs (12). The VFT module of most of the class C GPCRs contains the binding site of natural amino acids (glutamate, leucine, isoleucine, valine, etc.) or derivatives (like GABA), represented by an eight-residue motif that participates directly or indirectly in the binding of the α-amino acid functions (primary amine and carboxylic acid) (23). Among these eight residues, the serine shown to bind the α-COOH group of glutamate in mGluR1 (Ser_165_), and which is the most conserved residue in class C GPCRs, is strictly conserved in VKRs (except however in cestode and lepidopteran receptors). Other residues that bind the primary amine of the glutamate ligand in mGluR1 (Thr_188_, Asp_208_, Tyr_236_, and Asp_318_) are less conserved in VKRs, and the conserved residue Lys_409_ in mGluR1 responsible for the glutamate ligand binding is replaced by a conserved Tyr in VKRs (12). These data first indicate that the ligand of VKR is not glutamate, but very likely an amino acid molecule. Effectively, further work has demonstrated that arginine is the most potent amino acid able to activate many VKRs at 1 μM concentration and that arginine binding requires the presence of the essential Ser residue in the VFT module of these VKRs (17, 22).

**Figure 2:**
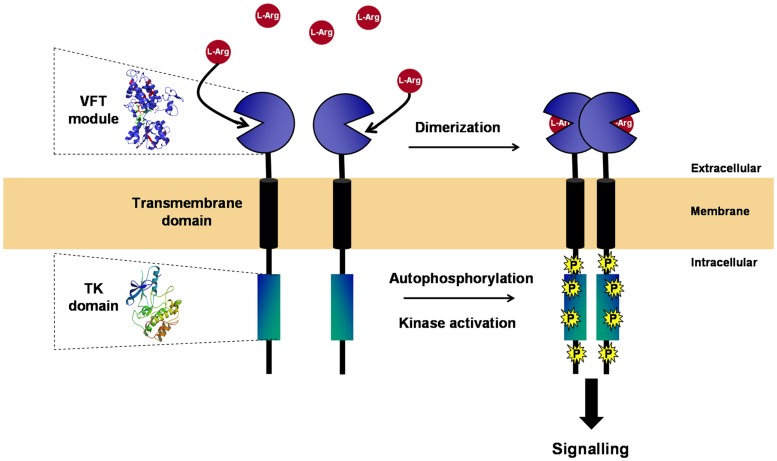
**Structure and activation of VKR**. VKR is composed of an intracellular TK domain similar to that of insulin receptor linked by a unique transmembrane domain to an extracellular moiety containing a venus fly trap (VFT) module. VFT domains are formed by two lobes separated by a highly flexible cleft, allowing the binding of a small molecule. VFT module of VKR was shown to bind amino acids and to have a high affinity for l-arginine. As many other RTKs, VKR is active as a dimer. Receptor dimerization occurs in the presence of l-Arg and leads to kinase activation, receptor autophosphorylation, and signal transduction.

With the exception of IR/IGFR receptors, which are disulfide-linked covalent dimers made of two extracellular α subunits and two transmembrane β subunits, all other RTKs are non-covalent dimers or oligomers in the activated state (24, 25). Dimerization of RTKs is required for activation, and similarly, it was clearly established that the VFT modules of the class C GPCRs and ANFR are functioning as dimers (26, 27). A three-dimensional model of the VKR of *Apis mellifera* was built, which suggested that a VFT dimer interface is present in VKR proteins similar to that in GPCRs. From time-resolved FRET measurements using SNAP-tag technology, it was demonstrated that honey bee VKR proteins expressed in HEK293T cells effectively constitute dimers at the cell surface (12). More recently, *S. mansoni* VKRs (SmVKR1 and SmVKR2) were shown to function also as homodimers when expressed in *Xenopus* oocytes. Receptor dimerization occurs in the presence of ligands and leads to kinase activation and receptor autophosphorylation (Figure 2). SmVKR1 and SmVKR2 were also shown to form active heterodimers (22).

## Potential Roles of VKR in Development and Reproduction

Quantitative RT-PCR performed on various developmental stages of the insects *A. mellifera* and *Tribolium castaneum* have indicated that *VKR* transcripts are much more abundant in larval stages than in others (nympha and imago). Furthermore, experiments indicated that transcription of *VKR* genes was particularly active in gonad tissues of the mosquito *Anopheles gambiae* and of the sea urchin *S. purpuratus* compared to the rest of the body. These results argued for a role of VKR proteins in embryonic and larval development as well as in reproduction (12). Recent studies performed on the parasite trematode *S. mansoni* have confirmed the importance of SmVKR receptors in development and reproduction. Both genes *Smvkr1* and *Smvkr2* are expressed in all stages of the parasite, with higher expression levels in larval stages (miracidium, sporocyst, and cercaria) (17). Immunolocalization experiments also showed the expression of SmVKR1 in miracidiums and sporocysts in proliferative germinal cells (15), which are similar to planarian neoblasts (28). Comparative analyses in adult parasites also indicated that expression of both *Smvkr1* and *Smvkr2* is higher in female worms than in males (17). By *in situ* hybridization, transcripts were detected in testes and more abundantly in ovaries, in which the expression profile of each *VKR* was noticeably different. *Smvkr1* transcripts were mainly present in the posterior part of the ovary that contains mature oocytes (in prophase I of meiosis) whereas *Smvkr2* transcripts were found in the anterior part of organ containing immature oocytes. Gene silencing of schistosome VKRs by RNA interference led to an important disorganization of the anteroposterior structure of the ovary and the knock down of *Smvkr1* resulted in the accumulation of big oocytes in the ovary and the absence of egg formation. In male testes, silencing of both *Smvkr* resulted in decrease of cell density within testicular lobes and in paucity of semen (22). These results highlighted the implication of VKRs in oogenesis and spermatogenesis in schistosomes, and overall confirmed their potential importance in reproduction processes in invertebrates.

## Prospects in VKR Signaling

If substantial knowledge has been already obtained about tissue expression and structure/activation of VKRs, not much is known to date about the signaling pathways and the cellular processes these uncommon receptors are susceptible to elicit. Recently, molecular partners of SmVKRs have been identified following a yeast two-hybrid (Y2H) screening of a schistosome cDNA library with active intracellular domains of VKRs as baits, and their characterization should help in further understanding of cellular and biological functions of VKRs. Interacting proteins were classified into five groups, according to putative functions in cytoskeleton reorganization, in vesicular trafficking, in kinase signaling, in gene expression, and in protein synthesis. Some of the VKR partners are already known for their roles in reproduction (22).

SmVKRs trapped a prefoldin subunit, a protein shown to be overexpressed in the ovary of schistosomes (29). Interestingly, prefoldin subunit 1 (pfd-1) mutant *C. elegans* worms develop abnormalities in gonadogenesis including oocyte migration defects (30). SmVKR1 also interacted with the Rho GTPase SmRho1 and the Zyxin/Trip6 adapter protein. SmRho1 is the homolog of RhoA, which was found to be involved in ovulation in *C. elegans*. It is expressed in *S. mansoni* gonads, in which it interacts with the Diaphanous homolog SmDia, suggesting its function in cytoskeleton organization (31). Zyxin is an LIM domain-containing protein known for its ability to shuttle between focal adhesions and nucleus, therefore influencing cell motility and gene transcription (32). Also, Zyxin was found to interact with germline RNA helicases GLH in P granules in *C. elegans* (33).

Among the SmVKR partners identified, several of them are nuclear proteins regulating gene expression. Their trapping might be explained by the recent finding that RTKs (such as ErbB2, ErbB4, VEGFR2, or IRs) are able to translocate to nucleus following internalization and to modulate gene expression (34). Human IR and IGF1-R translocation in nucleolar and perinucleolar areas is dependent on sumoylation on three lysine residues (K_1025_, K_1100_, and K_1120_) highly conserved in TK domains (35, 36) and interestingly, these residues are conserved in both SmVKRs. The interaction of two nucleolar proteins, Sirtuin 7 and tRNA d(2)-isopentenylpyrophosphatase, with both SmVKRs, is consistent with a possible nucleolar translocation of VKRs. Other nuclear proteins such as prp39, plac8, and ARID2 were found to interact with SmVKR1, reinforcing the idea of a role of VKRs in gene expression. Finally, SmVKR1 interacted with Notch, a key regulator of germline proliferation and meiosis progression (37–40).

**Figure 3:**
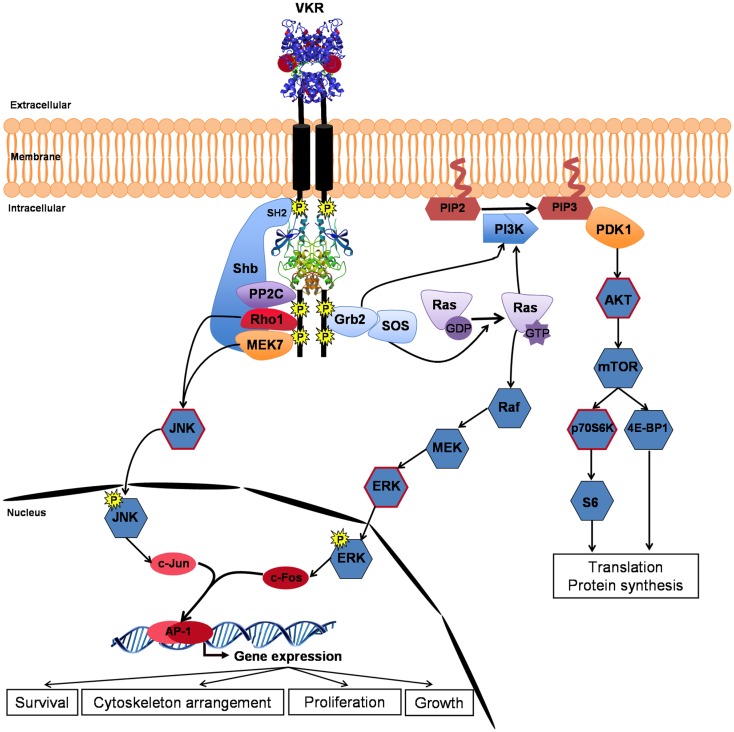
**VKR signaling pathways**. Closure of VFT extracellular domains upon binding of arginine ligand promotes and reinforces receptor dimerization inducing kinase activation and autophosphorylation of VKR. Phosphotyrosines can bind different partners for the transduction of conserved RTK signaling pathways, such as the PI3K/AKT/mTOR pathway involved in protein synthesis and the Ras/MAPK ERK pathway important for cell growth and proliferation. Moreover, activated VKRs could activate through the specific binding of Shb (SH2-containing protein), the alternative JNK pathway to contribute in concert with Rho1, MEK7, and PP2C to cytoskeleton rearrangement and oocyte maturation. Phosphorylation of AKT, p70S6K, ERK, and JNK (circled in red) was confirmed in VKR-expressing *Xenopus* oocytes (22).

As expected, several partners of VKR were proteins involved in phospho-signaling and the major pathways susceptible to be induced by activated VKRs are described in Figure 3. The SH2 domain-containing adapter Shb, previously known to bind RTK, was trapped by SmVKR1. Two other proteins, the protein phosphatase PP2C gamma and the MEK7 kinase, which are both involved in JNK activation pathways, were shown to interact with SmVKR1 specifically, as did the Shb protein, arguing for an implication of SmVKR1 in JNK/SAPK signaling (22).

Assumptions made about the nature of VKR phospho-pathways were comforted by analyses of VKR signaling in *Xenopus* oocytes. In this cellular model, ligand-activated RTKs generally elicit MAPK and PI3K/Akt/mTOR pathways. Similarly, it was shown that ligand-activated VKRs induce the phosphorylation of Erk1/2, Akt, and p70S6K, indicating that VKRs were susceptible to stimulate protein synthesis and cellular growth. Concerning the two other MAPK pathways, the JNK pathway was shown to be phosphorylated by VKRs but the p38γ/SAPK3 was not activated. JNK was phosphorylated in SmVKR1-expressing oocytes, corroborating the results of Y2H screening and the finding that SmVKR1 interacted with Rho1, Mek7, and PP2C (22). The JNK pathway was already shown to play an important role in oogenesis and meiosis resumption in *C. elegans* (33), in *D. melanogaster* (41), and in mammals (42, 43), and it is postulated that it could be used as a major pathway by VKRs in other invertebrates, including *S. mansoni* parasites, to influence oocyte maturation.

## VKRs as Potential Drug Targets

Venus kinase receptors are present in a large variety of invertebrates including many species of economical and medical importance. Their presence in gonads and their implication in gametogenesis and reproduction processes are valuable reasons to consider VKRs as interesting targets in control programs aiming at eradication of pests (like the red flour beetle *T. castaneum*), of vectors of infectious and parasitic diseases (like the mosquitoes *A. gambiae* vector of malaria*, Aedes aegypti* vector of dengue) or at the elimination of parasites (like *Schistosoma* and *Clonorchis* or other trematode and cestode species). In schistosomiasis, the second most important parasitic disease after malaria (44), the important fecundity of adult parasites is responsible for active schistosome transmission, but primarily the eggs are the cause of serious pathological disorders due to their trapping in the tissues (liver) of infected humans (45). Several TK inhibitors have been tested for their ability to affect the viability of worms and/or to decrease egg production, and their efficacy on the parasites has confirmed the main role played by TKs in schistosome reproduction processes (46, 47). Taking advantage of the similarity between the catalytic domains of *S. mansoni* IRs (SmIRs) and VKRs, a single drug was used to fight parasites by simultaneously targeting these receptors (48). Among various commercial RTK inhibitors active on mammal kinases, tyrphostin AG1024, with a well-established inhibitory effect on human IR/IGFR kinase (IC50 of about 5–10 μM) (49), was found to be the most potent inhibitory compound toward SmIR and SmVKR kinases. At micromolar doses, this drug induced apoptosis and caused death of larval parasites. In adult worms, AG1024 provoked alterations of reproductive organs, stopped egg laying and induced death of parasites, confirming again in this invertebrate model, the important role played by VKRs in reproduction processes (48). These results showed the possibility to use VKRs as novel targets in the control of schistosomes, and probably in other parasites and invertebrates, with the main advantage in the case of human parasites that they would be absent from the host kinase panel. Alternatively, the use of small molecules as antagonist ligands of the VFT domains of VKRs should be efficient to interfere specifically with VKR pathways and reproduction of organisms.

## Conclusion and Perspectives for Studies of VKR in Other Models

Venus kinase receptors constitute a novel class of RTKs for which limited knowledge remains available. Sequence similarity observed between their catalytic domains and those of IR and IR-like receptors was comforted by the demonstration of their great sensitivity to IR/IGFR kinase inhibitors (as tyrphostin AG1024). Moreover, as IRs and other growth factor receptors, VKRs, were shown to activate signaling pathways involved in metabolism and growth, such as PI3K/Akt and MAPK pathways, and the importance of VKRs in the biology of schistosome reproduction was demonstrated by RNAi gene silencing. MAPKs (ERK, p38, JNK) have evolved to transduce environmental and developmental signals (growth factors, stress) into adaptive and programed responses (differentiation, inflammation, apoptosis). Particularly, JNK is activated by diverse cellular stresses and it regulates many biological processes including apoptosis, cytoskeletal rearrangement, and also proliferation (50). Interestingly, the activation of JNK by VKRs, demonstrated in the schistosome model, might be related to the function of these receptors in gamete differentiation and maturation.

Considerable efforts have been made during the last years to develop molecular tools efficient for studying schistosome biology (RNAi, organ isolation, fluorescent labeling) (51–53) but in such parasite organisms, the complex life cycle and the uncapacity to propagate them *in vitro* represent major brakes to reverse genetics and in-depth fundamental studies. Planarians, like *Schmidtea mediterranea* and *Dugesia* species, which are free-living platyhelminths that also possess *VKR* genes, thus appear as attractive and alternative models to fill this gap (54). Indeed, planarian models are offering a large panel of molecular tools (including stem cell tracking, RNAi and whole-mount *in situ* hybridization) and they are famous for their incredible capacity for regeneration, being able to restore the whole body from a small part. Although planarians are hermaphrodites, their reproductive system shares common features with that of schistosomes (ovary and sperm ultrastructure, meiosis progression, yolk cells, etc.) (55), and it can be expected that along regeneration and RNAi protocols, they will bring in a near future essential information about the functions of VKR in gametogenesis and in development. Besides planarians, the mosquitoes *A. gambiae* and *A. aegypti*, in which insulin pathways, and possibly also VKR, are critical regulators of ovary growth and egg production (56), could also represent interesting models to study the function of VKR in reproduction. Both species allow the use of a wide range of molecular tools including transgenesis, organ isolation, immunofluorescence, and RNA interference and insightful works already demonstrated the possibility to study RTK functions in these species (57, 58). Finally, the sea urchin *S. purpuratus*, which presents external fertilization and embryogenesis, could offer the possibility to study the role of VKRs in embryo development by microinjection of RNA and/or morpholinos as previously described (59), a procedure that is not applicable to embryos of schistosomes due to the robust protein shell that surrounds eggs (60).

## Conflict of Interest Statement

The authors declare that the research was conducted in the absence of any commercial or financial relationships that could be construed as a potential conflict of interest.
